# Fabrication and Cytotoxicity of Fucoidan-Cisplatin Nanoparticles for Macrophage and Tumor Cells

**DOI:** 10.3390/ma10030291

**Published:** 2017-03-14

**Authors:** Pai-An Hwang, Xiao-Zhen Lin, Ko-Liang Kuo, Fu-Yin Hsu

**Affiliations:** 1 Department of Bioscience and Biotechnology, National Taiwan Ocean University, No. 2, Beining Road, Keelung City 202, Taiwan; a0985201229@gmail.com; 2 Seafood Technology Division, Council of Agriculture Fisheries Research Institute, No. 199 Hou-Ih Road, Keelung City 202, Taiwan; klkuo@mail.tfrin.gov.tw

**Keywords:** fucoidan, cisplatin, immune protection, anti-tumor activity

## Abstract

Fucoidan, an anionic, sulfated polysaccharide from brown seaweed, is known to exhibit antitumor and immunomodulatory functions. To develop an immune protection and chemotherapeutic agent, fucoidan-cisplatin nanoparticles (FCNPs) were designed. FCNPs were prepared by mixing cisplatin with fucoidan solution or fucoidan with cisplatin solution, followed by dialysis to remove trace elements. The nanoparticles, comprising 10 mg of fucoidan and 2 mg of cisplatin, which exhibited the highest cisplatin content and loading efficiency during the production process, were named as Fu100Cis20. The cisplatin content, cisplatin loading efficiency, nanoparticle size, and zeta potential of Fu100Cis20 were 18.9% ± 2.7%, 93.3% ± 7.8%, 181.2 ± 21.0 nm, and −67.4 ± 2.3 mV, respectively. Immune protection assay revealed that Fu100Cis20-treated RAW264.7 cells were protected from the cytotoxicity of cisplatin. Furthermore, antitumor assay indicated that Fu100Cis20-treated HCT-8 cells showed stronger cytotoxicity than those treated with cisplatin alone. These results suggested that fucoidan-based nanoparticles exhibited suitable particle size and high drug encapsulation, and that Fu100Cis20 has potential application in both immunotherapy and chemotherapy.

## 1. Introduction

Among thousands of platinum complexes that were synthesized and evaluated for their antitumor activity in the late 1970s, one of the most successful antitumor compound is *cis*-diaminedichloroplatinum (II), [Pt(NH_3_)_2_Cl_2_], clinically called cisplatin [1]. The antitumor mechanism of cisplatin is the induction of cytotoxicity by interference with transcription and/or DNA replication, and cisplatin crosslinks DNA in several different ways, interfering with cell division by mitosis. The damaged DNA elicits DNA repair mechanisms, which in turn activate apoptosis when repair proves impossible [2]. Moreover, cisplatin damages tumors via induction of apoptosis, mediation of calcium signaling, death receptor signaling, and activation of mitochondrial pathways [3]. Pruefer et al. demonstrated that cisplatin induces apoptosis in human colon cancer cells through the mitochondrial serine protease Omi/Htra2 [4]. However, conventional chemotherapy using cisplatin is not an effective method for the treatment of colorectal cancer clinically, owing to the low effective concentration of the drug that reaches the cancer site [5]. Therefore, colon-targeted drug delivery systems have been developed to improve the low utility rate of anticancer drugs [6]. Furthermore, clinical use of cisplatin is also limited by its severe toxic side effects; cisplatin is known to induce pro-inflammatory cytokines production and NF-κB activation, as well as increase the production of nitric oxide in macrophages in vitro [7]. Besides, a twofold increase in kidney macrophages has been observed in vivo after cisplatin administration [8], which could contribute to an inflammatory response in the kidney and may cause acute nephrotoxicity and chronic neurotoxicity [9]. Hence, approaches to reduce the toxic side effects of cisplatin and target cisplatin to the disease site are important issues in the development of effective drug delivery carriers.

Nanoscale drug carriers, such as liposomes and polymeric nanoparticles, present more efficient and safer delivery, reducing the side effects of the drugs. In tumor therapy, nanoparticles can enhance the permeability and retention effect caused by leaky tumor for better drug accumulation at the tumor sites [10]. These benefits make therapeutic nanoparticles a promising candidate that can replace traditional chemotherapy. Several polymers such as poly-γ glutamic acid (γ-PGA) [11] and polylactic acid [12] have been used as reservoirs for the delivery of cisplatin. In the present study, fucoidan, a class of sulfated and fucosylated polysaccharide extracted from brown seaweed [13], was selected as the raw material for the preparation of cisplatin carrier because of its biocompatibility [14] and bioactivities [15].

Fucoidan is an anionic sulfated polysaccharide, which exhibits multiple bioactivities, including antitumor [16,17,18], anti-inflammatory [19,20], anticoagulant [21], and enhancing osteogenic differentiation activities [22]. A number of studies have reported that fucoidan induces apoptosis to eliminate tumor cells [23,24]. Nevertheless, the biomedical applications of fucoidan-based nanoparticles encapsulating antitumor drugs are still at the early stage of development. Some studies have used fucoidan-based nanoparticles for encapsulating antitumor drugs such as curcumin [25] and doxorubicin [26]. The present study is the first to combine fucoidan-cisplatin into nanoparticles, evaluate the production process, and characterize the nanoparticles. In addition, the cell viability of normal cells (RAW264.7) and tumor cells (HCT-8) treated with the fucoidan-cisplatin nanoparticles (FCNPs) was also investigated.

## 2. Materials and Methods

### 2.1. Materials

Fucoidan isolated from *Fucus vesiculosus* (CAS number 9072-19-9, molecular weight range: 20,000–200,000) and cisplatin [*cis*-diammineplatinum (II) dichloride, Pt(NH_3_)_2_Cl_2_] were purchased from Sigma Aldrich^®^ (St. Louis, MO, USA). All other chemicals used were reagent grade.

### 2.2. Preparation of Fucoidan-Cisplatin Nanoparticles (FCNPs)

Two processes were used in this study for preparing FCNPs. Two milligrams cisplatin and 2.5, 5.0, 7.5 and 10.0 mg fucoidan were respectively dissolved in 1 mL deionized water, and 10 mg fucoidan and 0.5, 1.5, 2.0 and 4.0 mg cisplatin were respectively dissolved in 1 mL deionized water. The solution was gently stirred for three days under dark conditions. The solution was dialyzed against deionized water for 24 h at room temperature to remove unbound cisplatin (dialysis tube molecular weight cutoff = 3500). Finally, the dialyzed solution was lyophilized and named as FCNPs. The recovery (%) of FCNPs was determined as [FCNPs yield weight (mg)/Input weight (g)] × 100.

The content and loading efficiency of cisplatin incorporated into the FCNPs was determined using the *o*-phenylenediamine method [27]. Briefly, 0.1 mL of cisplatin-containing solution was mixed with 0.1 mL of 1,2-phenylenediamine solution in *N*,*N*-dimethylformamide (1.4 mg/mL) and 0.2 mL of KH_2_PO_4_ buffer solution (0.1 M, pH 6.8). The mixture was incubated at 100 °C for 4 min followed by absorbance measurement at 703 nm.

The cisplatin contents and loading efficiency were calculated as follows:

Cisplatin content (%) = (Amount of cisplatin liberated from the FCNPs/weight of FCNPs) × 100;

Cisplatin loading efficiency (%) = (Amount of cisplatin liberated fromthe FCNPs/feeding amount of cisplatin) × 100.

### 2.3. Characterization of Fucoidan-Cisplatin Nanoparticles

The lyophilized FCNPs were redistributed in deionized water to analyze the zeta potential and average particle size using a Zetasizer ZS (Malvern Instruments, Malvern, UK). The atomic composition of HCNPs was determined by energy-dispersive spectrometry (EDS) coupled to a transmission electron microscopy (TEM) (Hitachi H-600, Tokyo, Japan).

### 2.4. Cell Culture

RAW 264.7 cells (ATCC No. TIB-71), the mouse macrophage cell line, were obtained from the American Type Culture Collection. RAW 264.7 cells were cultured in Dulbecco’s modified Eagle medium (DMEM) supplemented with 10% heat-inactivated fetal bovine serum (FBS), penicillinG (100 U/mL), and streptomycin (100 μg/mL), and cells were maintained at 37 °C in an atmosphere of 5% CO_2_. HCT-8 cells (ATCC No. CCL-224), the human ileocecal adenocarcinoma tumor cells, were obtained from the American Type Culture Collection. Cells were cultured in RPMI 1640 medium (Sigma Chemical Co., St. Louis, MO, USA) supplemented with 10% FBS, 100 U of penicillin per mL, 100 g of streptomycin per mL, and 0.25 g of amphotericin B per mL, and cells were maintained at 37 °C in an atmosphere of 5% CO_2_.

### 2.5. Cells Viability of Cisplatin, Fucoidan, and Fu100Cis20 in RAW264.7 and HCT-8 Cells

Fu100Cis20 nanoparticle was made from 10 mg fucoidan and 2 mg cisplatin. After conversion to cisplatin content (18.9%), 0.02, 0.20 and 0.40 mg/mL of Fu100Cis20 contained 3.78, 37.8 and 75.6 μg/mL of cisplatin, therefore we took 4, 40 and 80 μg/mL cisplatin to treat with RAW 264.7 macrophage cells for 24 h for cell viability assay. Similarly, 0.25, 0.50 and 1.00 mg/mL of Fu100Cis20 contained 47.25, 94.5 and 189 μg/mL of cisplatin, and 50, 100 and 200 μg/mL cisplatin treated with HCT-8 tumor cells for 24 h for cell viability assay. Fucoidan and Fu100Cis20 were used in the same dose in this study. In cell viability assay, cells (2 × 10^5^ per well) were treated with samples and without samples under normal cell culture condition. Proliferation of cells was determined by streptomycin and the 3-(4,5-dimethylthiazol-2-yl)-2,5-diphenyltetrazolium bromide (MTT) colorimetric assay. Cells were reacted with MTT (1 mg/mL) for 4 h, and absorbance readouts were recorded at 570 nm by an enzyme-linked immunosorbent assay (ELISA) reader (Molecular Devices, Tokyo, Japan). The percent relative activity was determined as (A1/A0) 100%, where A0 and A1 are the absorbances in the absence of samples and presence of samples, respectively.

### 2.6. Statistical Analysis

Statistical analysis of the data was performed using nonparametric Kruskal-Wallis test and the posthoc Tukey’s multiple comparison tests, by Graph Pad Prism 5.0 (GraphPad Software, La Jolla, CA, USA). Differences between the groups with *p* < 0.05 were considered statistically significant.

## 3. Results and Discussion

### 3.1. Production Process of FCNPs

Fucoidan has hydrophilic surface groups, such as sulfated, hydroxyl, and carboxyl groups, which can interact with other atoms to form nanoparticles [25,26,28]. In our previous study, we demonstrated that the interaction of cisplatin, a platinum (II) drug, with anionic polysaccharide could cause spontaneous folding to form a nanoparticles [29], similar to the metal-anion polymer complexes reported by Nishiyama et al. [30]. In the present study, we used fucoidan, an anionic polysaccharide, to develop nanoparticles for cisplatin tumor drug delivery.

To determine the optimal FCNPs production process, two processes were employed. In process I, 2.5, 5.0, 7.5 and 10.0 mg of fucoidan were respectively dissolved in 1 mL of deionized water and mixed with 2 mg of cisplatin to obtain the lyophilized products Fu25Cis20, Fu50Cis20, Fu75Cis20, and Fu100Cis20, respectively. As shown in Table 1, the recovery of Fu75Cis20 (84.2% ± 4.7%) and Fu100Cis20 (82.8 ± 5.7) was higher than that of Fu25Cis20 (62.2 ± 5.5) and Fu50Cis20 (72.6 ± 0.3), and the cisplatin content and loading efficiency of Fu100Cis20 (18.9% ± 2.7% and 93.3% ± 7.8%) were higher than those of the other products. In process II, 10 mg of fucoidan were mixed with 0.5, 1.5, 2.0 and 4.0 mg of cisplatin to obtain the lyophilized products Fu100Cis5, Fu100Cis15, Fu100Cis20, and Fu100Cis40, respectively. It was found that the recovery, cisplatin content, and loading efficiency of Fu100Cis20 were higher than those of the other products, and that Fu100Cis5 exhibited the lowest cisplatin content (3.4% ± 0.3%) and Fu100Cis40 presented the lowest cisplatin loading efficiency (26.2% ± 1.2%) (Table 2). Based on the results illustrated in Table 1 and Table 2, it can be concluded that with the increasing proportion of fucoidan, more cisplatin was complxed (e.g., Fu100Cis20). However, when the cisplatin loading content was higher than the fucoidan encapsulating ability, the cisplatin loading efficiency significantly decreased (e.g., Fu100Cis40). In a previous study, Cai et al. [31] demonstrated that up to 0.75 w/w cisplatin-hyaluronan was obtained with decreasing cisplatin content in a cisplatin-hyaluronan nanoparticles production process. Furthermore, curcumin-fucoidan nanoparticles were used as a natural antitumor drug, and its loading efficiency was higher than 85% [25]. In addition, nanoparticles comprising doxorubicin, an antitumor drug, and fucoidan were used in tumor therapy, and its drug loading efficiency and drug content were 71.1% and 3.6%, respectively [26].

### 3.2. Characteristics of FCNPs and Fu100Cis20

Size variability is one of the advantages of nanoparticles in antitumor therapy because the nanoparticles can remain in the tumor microenvironment in which solid tumors are characterized by a leaky vasculature. The size of the gap junction between the endothelial cells of leaky tumor vasculature may range from 100 to 600 nm [32]. Accordingly, to achieve increased intratumoral distribution and accumulation for improved therapeutic response, the size of FCNPs should be between 100 and 600 nm. In the present study, the size and zeta potential of FCNPs developed using process I and II were determined. As shown in Table 3, the size of FCNPs developed from process I was in the range of approximately 181–468 nm, and Fu100Cis had the smallest size. The zeta potential of the FCNPs decreased from −31.5 ± 4.7 to −67.4 ± 2.3 mV with the increasing fucoidan loading content from 2.5 to 10 mg. With regard to the size of FCNPs developed from process II, it was narrower in the range of approximately 145–224 nm. Although Fu100Cis15 and Fu100Cis20 had similar size (193.4 ± 31.1 and 181.2 ± 21.0 nm, respectively), considering their cisplatin content (11.1% ± 0.4% and 18.9% ± 2.7%, respectively) and loading efficiency (66.1% ± 4.9% and 93.3% ± 7.8%, respectively) (Table 2), Fu100Cis20 was better with a zeta potential of −67.4 ± 2.3 mV (Table 4).

**Figure 1:**
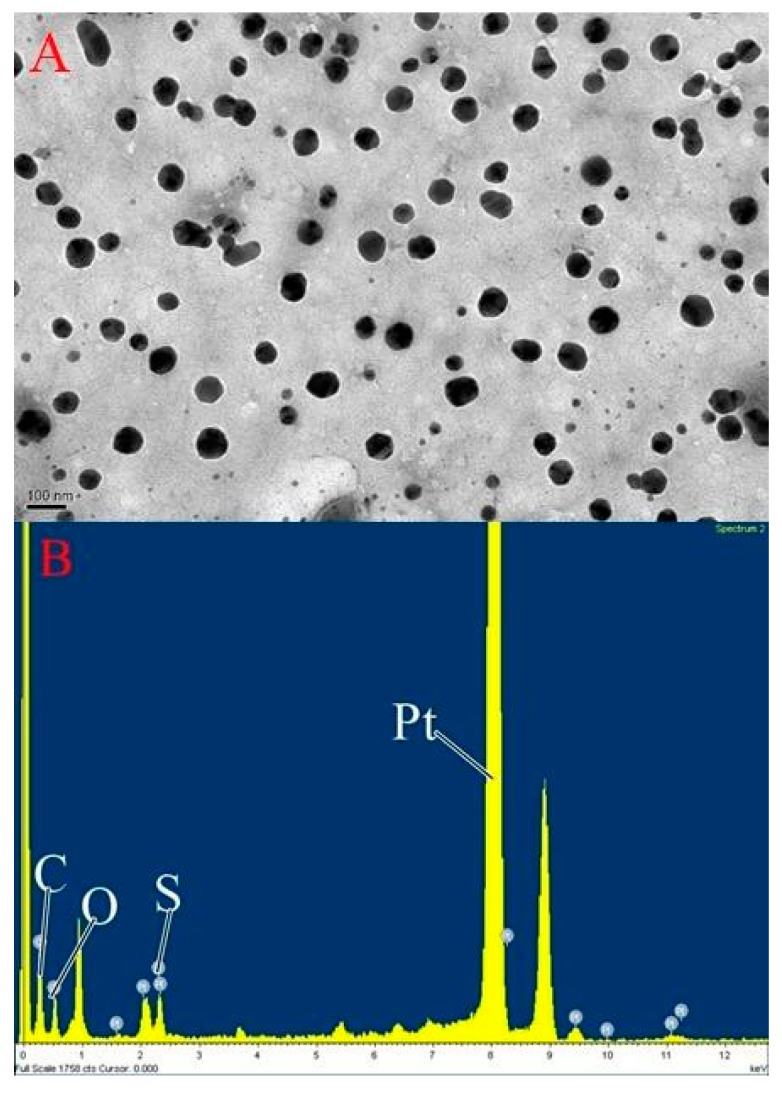
(**A**) Transmission electron microscopy (TEM) image of Fu100Cis20 nanoparticles; (**B**) Corresponding energy-dispersive X-ray spectroscopy spectrum.

Based on the results obtained (Table 1, Table 2, Table 3 and Table 4), the nanoparticles comprising 10 mg of fucoidan and 2 mg of cisplatin, which exhibited the highest cisplatin content and loading efficiency, were named as Fu100Cis20. The cisplatin content, cisplatin loading efficiency, nanoparticle size, and zeta potential of Fu100Cis20 were 18.9% ± 2.7%, 93.3% ± 7.8%, 181.2 ± 21.0 nm, and −67.4 ± 2.3 mV, respectively. It has been reported that the particle size of fucoidan nanoparticles for tumor drug should be in the range of approximately 100–274 nm [9,25,26,33]. The Fu100Cis20 nanoparticles were essentially spherical in shape (Figure 1A), with an average diameter of approximately 180 nm, which was consistent with the measurements by dynamic light scattering. The atomic composition of Fu100Cis20, as determined by TEM-EDS (Figure 1B), confirmed fucoidan-cisplatin nanoparticles containing fucoidan and cisplatin.

### 3.3. Immunomodulatory Activity of Cisplatin, Fucoidan, and Fu100Cis20

Maruyama et al. [34] reported that fucoidan exhibits antitumor activity by activating the immune cell activity and cytokine production. Furthermore, fucoidan can produce immune-stimulating effects on dendritic cells [35] and natural killer cells [36], and can enhance antitumor immunity through immune cell activation and stimulation of antitumor cytokines production. In contrast, cisplatin exerts its antitumor activity and cytotoxic effect by binding to genomic DNA in the cell nucleus, and its cytotoxic effect is non-specific. Thus, cisplatin may also induce immunological cell death [37]. Therefore, we used fucoidan for reducing the cytotoxicity of cisplatin in RAW264.7 macrophage cells and enhancing cytotoxicity of of cisplatin in HCT-8 tumor cells in the present study. First, we investigated the immunomodulatory activity of cisplatin, fucoidan, and Fu100Cis20 in RAW264.7 cells.

**Figure 2:**
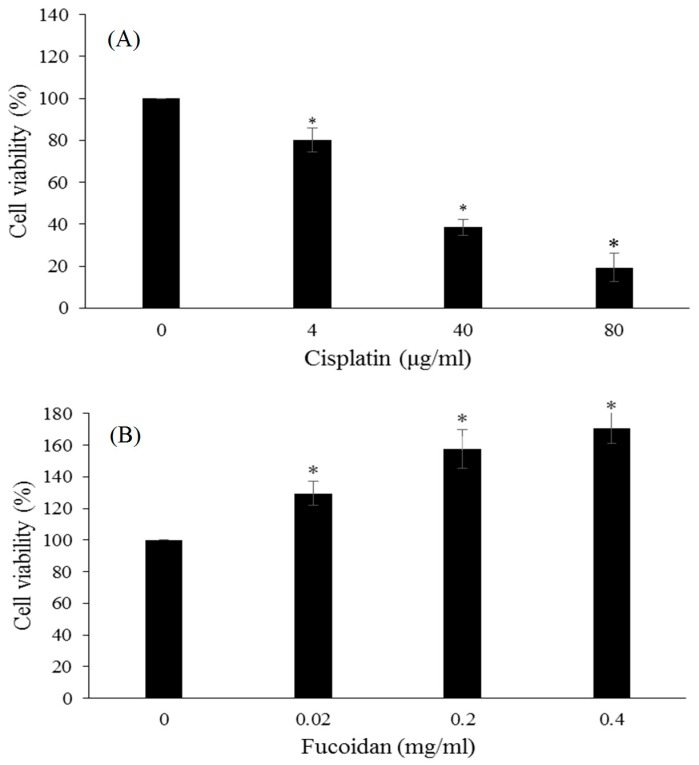

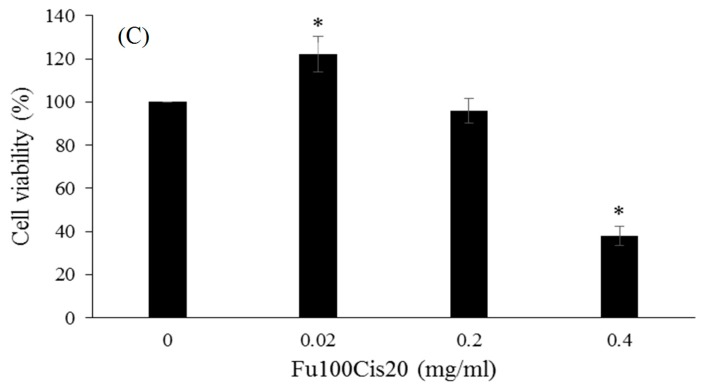
Cell viability results of (**A**) cisplatin; (**B**) fucoidan and (**C**) Fu100Cis20 in RAW264.7 cells for 24 h. Values were expressed as mean ± SD, *n* = 5. * *p* < 0.05 when compared with 0 mg/mL group.

After conversion to cisplatin content (18.9%) 0.02, 0.20 and 0.40 mg/mL of Fu100Cis20 contained 3.78, 37.8, and 75.6 μg/mL of cisplatin, here we took similar amount cisplatin, 4, 40 and 80 μg/mL, to treat with RAW 264.7 macrophage cells for determining cell viability assay. After treatment of RAW264.7 cells with cisplatin, the cell viability significantly decreased and strong cytotoxicity was observed (Figure 2A). In contrast, fucoidan treatment increased the cell viability of RAW264.7 cells and produced no cytotoxicity (Figure 2B). In Figure 2C, 0.2 mg Fu100Cis20 contained nearly 40 μg cisplatin, the cell viability of 0.2 mg/mL Fu100Cis20 and 40 μg/mL cisplatin were 96.10% ± 5.69% and 38.55% ± 3.90%, and 0.2 mg/mL Fu100Cis20 obtained less cytotoxicity than 40 μg/mL cisplatin RAW264.7 macrophage cells. Thus, we suggested that the cytotoxicity of cisplatin was reducing by fucoidan, and we demonstrated that Fu100Cis20 had lower cytotoxicity than cisplatin alone. Song et al. [38] reported that fucoidan can ameliorate cisplatin-induced gastrointestinal dysfunction. Accordingly, we suggested that fucoidan can form stable combined with cisplatin, and that fucoidan-cisplatin nanoparticles can reduce the cytotoxicity of cisplatin. Similarly, Jenog et al. [39] and Cohen et al. [40] proposed that polysaccharide-based nanoparticles can reduce systemic toxicity from drug.

### 3.4. Cytotoxicity against Tumor Cell of Cisplatin, Fucoidan and Fu100Cis20

Besides enhancing anticancer immunity, fucoidan can also exert antitumor activity by inducing apoptosis. Fucoidan has been reported to induce apoptosis in HT-29 and HCT116 human colon cancer cells via both death receptor-mediated and mitochondria-mediated apoptotic pathways [41]. Thus, Fu100Cis20 might have better antitumor effect than cisplatin.

**Figure 3:**
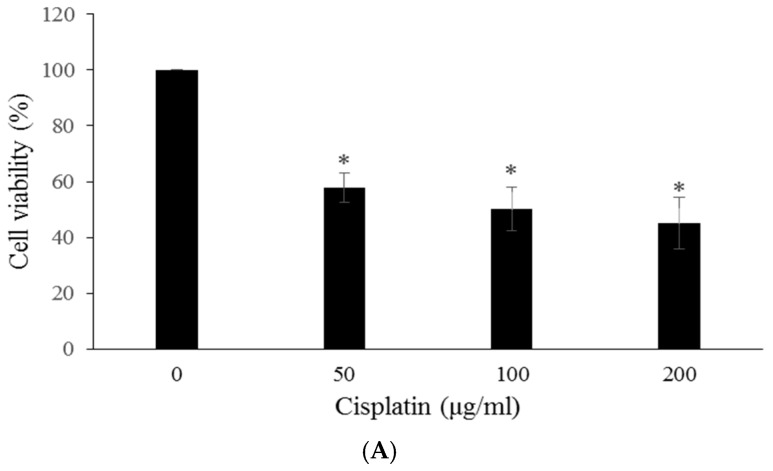

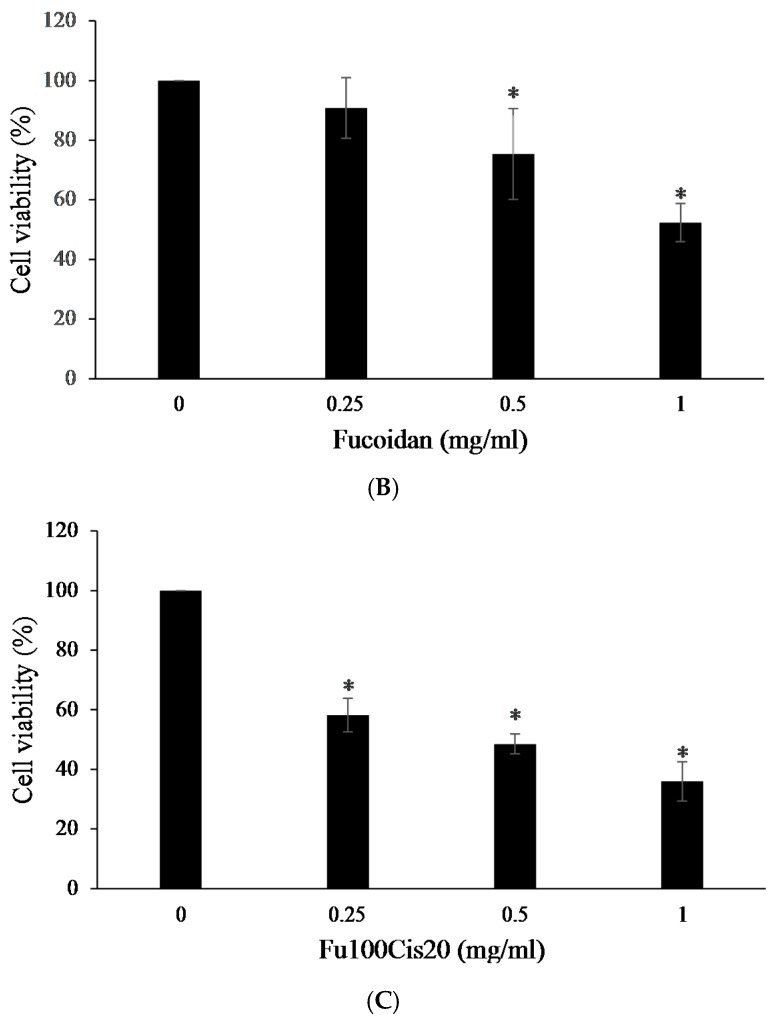
Cell viability results of (**A**) cisplatin; (**B**) fucoidan and (**C**) Fu100Cis20 in HCT-8 cells for 24 h. Values were expressed as mean ± SD, *n* = 5. * *p* < 0.05 when compared with 0 mg/mL group.

After conversion to cisplatin content (18.9%), 0.25, 0.50 and 1.00 mg/mL of Fu100Cis20 contained 47.25, 94.5, and 189 μg/mL of cisplatin, and we took 50, 100, and 200 μg/mL cisplatin treated with HCT-8 cells for cell viability assay. The results showed that 200 μg/mL cisplatin reduced the cell viability of HCT-8 cells to 45.12% ± 9.34% (Figure 3A), and 1 mg/mL fucoidan inhibited the growth of HCT-8 cells to 52.34% ± 6.38% (Figure 3B). However, 1 mg/mL Fu100Cis20, which contained approximately 200 μg/mL cisplatin, showed stronger cytotoxicity in HCT-8 cells (35.95% ± 6.60%) than 200 μg/mL cisplatin (Figure 3C). These results demonstrated that the cytotoxicity induced by Fu100Cis20 in HCT-8 cells was dose-dependent, and that Fu100Cis20 had greater cytotoxic effect on tumor cells than cisplatin. Thus, we suggested that the stronger cytotoxicity was offer by fucoidan, and we demonstrated that Fu100Cis20 had better cytotoxicity than cisplatin alone. In our present study, fucoidan-cisplatin nanoparticles were formed by metallic interaction, and the average particle size of Fu100Cis20 was very stable at room temperature, pH7.0, for one week (data not show). Thus, it was suggested that cisplatin was released from fucoidan-cisplatin nanoparticles at weak acidic environment of tumor tissues, and exhibited cytotoxicity to HCT-8 cells [42].

## 4. Conclusions

This study described a new colonic drug delivery system comprising fucoidan and cisplatin. To the best of our knowledge, this is the first report on the rapid synthesis of stable fucoidan-cisplatin nanoparticles that exhibit significant immune protection and antitumor activity. The production process of these nanoparticles is eco-friendly because no solvents or toxic chemicals are used, it is fast and simple, and it can easily be upgraded for large-scale industrial production.

## Figures and Tables

**Table 1:** Recovery, cisplatin content and cisplatin loading efficiency of fucoidan-cisplatin nanoparticles by 2 mg cisplatin mixing with different weights of fucoidan.

	Fucoidan (mg)	Cisplatin (mg)	Input Weight (mg)	Recovery (%)	Cisplatin Content (%)	Cisplatin Loading Efficiency (%)
Fu25Cis20	2.5	2.0	4.5	62.2 ± 5.5 a	6.9 ± 1.6 a	9.5 ± 1.4 a
Fu50Cis20	5.0	2.0	7.0	72.6 ± 0.3 b	11.1 ± 0.3 b	28.3 ± ± 2.3 b
Fu75Cis20	7.5	2.0	9.5	84.2 ± 4.7 c	13.4 ± 3.1 b	53.9 ± 13.1 c
Fu100Cis20	10.0	2.0	12.0	82.8 ± 5.7 c	18.9 ± 2.7 c	93.3 ± 7.8 d

Means with the same letter were not significantly different in post-hoc tests (*p* < 0.05).

**Table 2:** Recovery, cisplatin content and cisplatin loading efficiency of fucoidan-cisplatin nanoparticles by 10 mg fucoidan mixing with different weights of cisplatin.

	Fucoidan (mg)	Cisplatin (mg)	Input Weight (mg)	Recovery (%)	Cisplatin Content (%)	Cisplatin Loading Efficiency (%)
Fu100Cis5	10.0	0.5	4.5	85.7 ± 4.3 a	3.4 ± 0.3 a	59.5 ± 0.9 a
Fu100Cis15	10.0	1.5	7.0	74.8 ± 4.6 b	11.1 ± 0.4 b	66.1 ± ± 4.9 b
Fu100Cis20	10.0	2.0	12.0	82.8 ± 5.7 a	18.9 ± 2.7 c	93.3 ± 7.8 c
Fu100Cis40	10.0	4.0	14.0	75.0 ± 2.0 b	10.0 ± 0.2 b	26.2 ± 1.2 d

Means with the same letter were not significantly different in post-hoc tests (*p* < 0.05).

**Table 3:** Size and zeta potential of fucoidan-cisplatin nanoparticles by 2 mg cisplatin mixing with different weights of fucoidan.

	Fucoidan (mg)	Cisplatin (mg)	Average Particle Size (nm)	Zeta Potential (mV)
Fu25Cis20	2.5	2.0	468.1 ± 21.7 a	−31.5 ± 4.7 a
Fu50Cis20	5.0	2.0	426.2 ± 15.8 a	−33.5 ± 5.6 a
Fu75Cis20	7.5	2.0	391.6 ± 34.9 a	−44.9 ± 4.5 b
Fu100Cis20	10.0	2.0	181.2 ± 21.0 b	−67.4 ± 2.3 c

Means with the same letter were not significantly different in post-hoc tests (*p* < 0.05).

**Table 4:** Size and zeta potential of fucoidan-cisplatin nanoparticles by 10 mg fucoidan mixing with different weights of cisplatin.

	Fucoidan (mg)	Cisplatin (mg)	Average Particle Size (nm)	Zeta Potential (mV)
Fu100Cis5	10.0	0.5	224.0 ± 45.2 a	−63.2 ± 5.2 a
Fu100Cis15	10.0	1.5	193.4 ± 31.1 b	−68.9 ± 8.3 a
Fu100Cis20	10.0	2.0	181.2 ± 21.0 b	−67.4 ± 2.3 a
Fu100Cis40	10.0	4.0	145.2 ± 23.0 c	−59.4 ± 8.0 b

Means with the same letter were not significantly different in post-hoc tests (*p* < 0.05).

## References

[1] Gordon M., Hollander S. (1993). Review of platinum anticancer compounds. J. Med..

[2] Wang D., Lippard S.J. (2005). Cellular processing of platinum anticancer drugs. Nat. Rev. Drug Discov..

[3] Florea A.M., Büsselberg D. (2011). Cisplatin as an anti-tumor drug: Cellular mechanisms of activity, drug resistance and induced side effects. Cancers.

[4] Pruefer F.G., Lizarraga F., Maldonado V., Melendez-Zajgla J. (2008). Participation of Omi Htra2 serine-protease activity in the apoptosis induced by cisplatin on SW480 colon cancer cells. J. Chemother..

[5] Fink D., Nebel S., Aebi S., Zheng H., Cenni B., Nehme A., Christen R.D., Howell S.B. (1996). The role of DNA mismatch repair in platinum drug resistance. Cancer Res..

[6] Morral-Ruiz G., Melgar-Lesmes P., Solans C., Garcia-Celma M.J. (2013). Multifunctional polyurethane-urea nanoparticles to target and arrest inflamed vascular environment: A potential tool for cancer therapy and diagnosis. J. Control. Release.

[7] Chauhan P., Sodhi A., Shrivastava A. (2009). Cisplatin primes murine peritoneal macrophages for enhanced expression of nitric oxide, proinflammatory cytokines, TLRs, transcription factors and activation of MAP kinases upon co-incubation with L929 cells. Immunobiology.

[8] Lu L.H., Oh D.J., Dursun B., He Z., Hoke T.S., Faubel S., Edelstein C.L. (2008). Increased macrophage infiltration and fractalkine expression in cisplatin-induced acute renal failure in mice. J. Pharmacol. Exp. Ther..

[9] Pinheiro A.C., Bourbon A.I., Cerqueira M.A., Maricato E., Nunes C., Coimbra M.A., Vicente A.A. (2015). Chitosan/fucoidan multilayer nanocapsules as a vehicle for controlled release of bioactive compounds. Carbohydr. Polym..

[10] Greish K. (2010). Enhanced permeability and retention (EPR) effect for anticancer nanomedicine drug targeting. Methods Mol. Biol..

[11] Ye H., Jin L., Hu R., Yi Z., Li J., Wu Y., Xi X., Wu Z. (2006). Poly(gamma,l-glutamic acid)-cisplatin conjugate effectively inhibits human breast tumor xenografted in nude mice. Biomaterials.

[12] Araki H.T.T., Kodama M. (1999). Anti-tumor effect of cisplatin incorporated into polylactic acid microcapsules. Artif. Organs.

[13] Kylin H. (1913). Biochemistry of sea algae. Phys. Chem..

[14] Venkatesan J., Bhatnagar I., Kim S.K. (2014). Chitosan-Alginate Biocomposite Containing Fucoidan for Bone Tissue Engineering. Mar. Drugs.

[15] Chen M.C., Hsu W.L., Hwang P.A., Chen Y.L., Chou T.C. (2016). Combined administration of fucoidan ameliorates tumor and chemotherapy-induced skeletal muscle atrophy in bladder cancer-bearing mice. Oncotarget.

[16] Hsu H.Y., Lin T.Y., Hwang P.A., Tseng L.M., Chen R.H., Tsao S.M., Hsu J. (2013). Fucoidan induces changes in the epithelial to mesenchymal transition and decreases metastasis by enhancing ubiquitin-dependent TGFbeta receptor degradation in breast cancer. Carcinogenesis.

[17] Hsu H.Y., Lin T.Y., Wu Y.C., Tsao S.M., Hwang P.A., Shih Y.W., Hsu J. (2014). Fucoidan inhibition of lung cancer in vivo and in vitro : Role of the Smurf2-dependent ubiquitin proteasome pathway in TGFbeta receptor degradation. Oncotarget.

[18] Chen M.C., Hsu W.L., Hwang P.A., Chou T.C. (2015). Low Molecular Weight Fucoidan Inhibits Tumor Angiogenesis through Downregulation of HIF-1/VEGF Signaling under Hypoxia. Mar. Drugs.

[19] Hwang P.A., Chien S.Y., Chan Y.L., Lu M.K., Wu C.H., Kong Z.L., Wu C.J. (2011). Inhibition of Lipopolysaccharide (LPS)-induced inflammatory responses by Sargassum hemiphyllum sulfated polysaccharide extract in RAW 264.7 macrophage cells. J. Agric. Food Chem..

[20] Hwang P.A., Hung Y.L., Chien S.Y. (2015). Inhibitory activity of Sargassum hemiphyllum sulfated polysaccharide in arachidonic acid-induced animal models of inflammation. J. Food Drug Anal..

[21] Irhimeh M.R., Fitton J.H., Lowenthal R.M. (2009). Pilot clinical study to evaluate the anticoagulant activity of fucoidan. Blood Coagul. Fibrinolysis.

[22] Hwang P.A., Hung Y.L., Phan N.N., Hieu B.T.N., Chang P.M., Li K.L., Lin Y.C. (2016). The in vitro and in vivo effects of the low molecular weight fucoidan on the bone osteogenic differentiation properties. Cytotechnology.

[23] Chen S., Zhao Y., Zhang Y., Zhang D. (2014). Fucoidan induces cancer cell apoptosis by modulating the endoplasmic reticulum stress cascades. PLoS ONE.

[24] Park H.Y., Kim G.Y., Moon S.K., Kim W.J., Yoo Y.H., Choi Y.H. (2014). Fucoidan inhibits the proliferation of human urinary bladder cancer T24 cells by blocking cell cycle progression and inducing apoptosis. Molecules.

[25] Huang Y.C., Kuo T.H. (2016). O-carboxymethyl chitosan/fucoidan nanoparticles increase cellular curcumin uptake. Food Hydrocoll..

[26] Lee K.W., Jeong D., Na K. (2013). Doxorubicin loading fucoidan acetate nanoparticles for immune and chemotherapy in cancer treatment. Carbohydr. Polym..

[27] Golla E.D., Ayres G.H. (1973). Spectrophotometric determination of platinum with o-phenylenediamine. Talanta.

[28] Ermakova S., Kusaykin M., Trincone A., Tatiana Z. (2015). Are multifunctional marine polysaccharides a myth or reality?. Front. Chem..

[29] Tsai S.W., Yu D.S., Tsao S.W., Hsu F.Y. (2013). Hyaluronan-cisplatin conjugate nanoparticles embedded in Eudragit S100-coated pectin/alginate microbeads for colon drug delivery. Int. J. Nanomed..

[30] Nishiyama N., Okazaki S., Cabral H., Miyamoto M., Kato Y., Sugiyama Y., Nishio K., Matsumura Y., Kataoka K. (2003). Novel cisplatin-incorporated polymeric micelles can eradicate solid tumors in mice. Cancer Res..

[31] Cai S., Xie Y.M., Bagby T.R., Cohen M.S., Forrest M.L. (2008). Intralymphatic chemotherapy using a hyaluronan-cisplatin conjugate. J. Surg. Res..

[32] Yuan F., Dellian M., Fukumura D., Leunig M., Berk D.A., Torchilin V.P., Jain R.K. (1995). Vascular permeability in a human tumor xenograft: Molecular size dependence and cutoff size. Cancer Res..

[33] Huang Y.C., Lam U.I. (2011). Chitosan/Fucoidan pH Sensitive Nanoparticles for Oral Delivery System. J. Chin. Chem. Soc..

[34] Maruyama H., Tamauchi H., Iizuka M., Nakano T. (2006). The role of NK cells in antitumor activity of dietary fucoidan from Undaria pinnatifida sporophylls (Mekabu). Planta Med..

[35] Kim M.H., Joo H.G. (2008). Immunostimulatory effects of fucoidan on bone marrow-derived dendritic cells. Immunol. Lett..

[36] Ale M.T., Maruyama H., Tamauchi H., Mikkelsen J.D., Meyer A.S. (2011). Fucoidan from *Sargassum* sp. and Fucus vesiculosus reduces cell viability of lung carcinoma and melanoma cells in vitro and activates natural killer cells in mice in vivo. Int. J. Biol. Macromol..

[37] Mehmood R.K. (2014). Review of Cisplatin and oxaliplatin in current immunogenic and monoclonal antibody treatments. Oncol. Rev..

[38] Song M.Y., Ku S.K., Kim H.J., Han J.S. (2012). Low molecular weight fucoidan ameliorating the chronic cisplatin-induced delayed gastrointestinal motility in rats. Food Chem. Toxicol..

[39] Jeong Y.I., Kim S.T., Jin S.G., Ryu H.H., Jin Y.H., Jung T.Y., Kim I.Y., Jung S. (2008). Cisplatin-incorporated hyaluronic acid nanoparticles based on ion-complex formation. J. Pharm. Sci..

[40] Cohen M.S., Cai S., Xie Y., Forrest M.L. (2009). A novel intralymphatic nanocarrier delivery system for cisplatin therapy in breast cancer with improved tumor efficacy and lower systemic toxicity in vivo. Am. J. Surg..

[41] Kim E.J., Park S.Y., Lee J.Y., Park J.H. (2010). Fucoidan present in brown algae induces apoptosis of human colon cancer cells. BMC Gastroenterol..

[42] Fan X.H., Zhao X.S., Qu X.K., Fang J. (2015). pH sensitive polymeric complex of cisplatin with hyaluronic acid exhibits tumor-targeted delivery and improved in vivo antitumor effect. Int. J. Pharm..

